# Managing intraoperative rupture of internal carotid pseudoaneurysms during endoscopic transnasal optic canal decompression: a case report

**DOI:** 10.3389/fneur.2024.1382793

**Published:** 2024-06-19

**Authors:** Zeran Yu, Junhui Qi, Lei Wang, Xiang Yang, Zhengqiao Liu, Xu Chen, Hongling Xu, Yajie Li, Yuyun Chen, Chengguo Dai, Zhen Gu

**Affiliations:** ^1^Department of Neurosurgery, The Affiliated Hospital of Yunnan University, Second People’s Hospital of Yunnan, Kunming, China; ^2^Department of Neurosurgery, Beijing Tiantan Hospital Yunnan, Kunming, China; ^3^The Center of Stroke, The Affiliated Hospital of Yunnan University, Second People’s Hospital of Yunnan, Kunming, China; ^4^Department of Neurosurgery, Huashan Hospital, Fudan University, Shanghai, China; ^5^Department of Obstetrics, The First People’s Hospital of Yunnan, The Affiliated Hospital of Kunming University of Science and Technology, Kunming, China

**Keywords:** pseudoaneurysm, intraoperative hemorrhage, traumatic optic neuropathy, endoscopic transnasal optic canal decompression, endovascular embolization

## Abstract

**Background:**

Endoscopic transnasal optic canal decompression is widely used in the treatment of traumatic optic neuropathy (TON) following head and craniofacial trauma. Intraoperative hemorrhage is a catastrophic surgical complication during optic canal decompression.

**Case description:**

We present two cases of patients with TON who suffered unexpected intra-operative massive bleeding during endoscopic transnasal optic canal decompression. After intraoperative hemostasis was achieved, emergent cerebral angiograms demonstrated the formation of internal carotid pseudoaneurysms, which were immediately embolized with coils combined with or without Onyx with balloon assistance. One of these cases was also complicated by a postoperative cerebrospinal fluid leak, which failed to be treated with lumbar drainage but was successfully repaired with endoscopic transnasal surgery.

**Conclusion:**

The intra-operative rupture of ICA pseudoaneurysm is a rare but catastrophic complication in TON patients. Intraoperative massive bleeding indicates rupture of ICA pseudoaneurysm. Postoperative emergency angiography and endovascular therapy should be arranged to evaluate and repair the cerebral vascular injury. Endoscopic trans-nasal surgery repairing CSF leaks resistant to lumbar drainage could be efficient and safe following pseudoaneurysm embolization.

## Introduction

Traumatic optic neuropathy (TON), caused by head and craniofacial trauma, manifests partial or complete visual loss (1). Endoscopic transnasal optic canal decompression is a popular surgical technique for treating TON (1, 2). In addition, skull base fracture following head trauma often leads to internal carotid artery injury, including traumatic pseudoaneurysms, carotid artery dissection, and carotid cavernous fistula. We present two cases of patients with TON who suffered intra-operative massive bleeding during endoscopic transnasal optic canal decompression. An emergent cerebral angiogram after intraoperative hemostasis demonstrated the formation of internal carotid pseudoaneurysms, which were immediately embolized with coils under balloon assistance.

## Case 1

**Figure 1:**
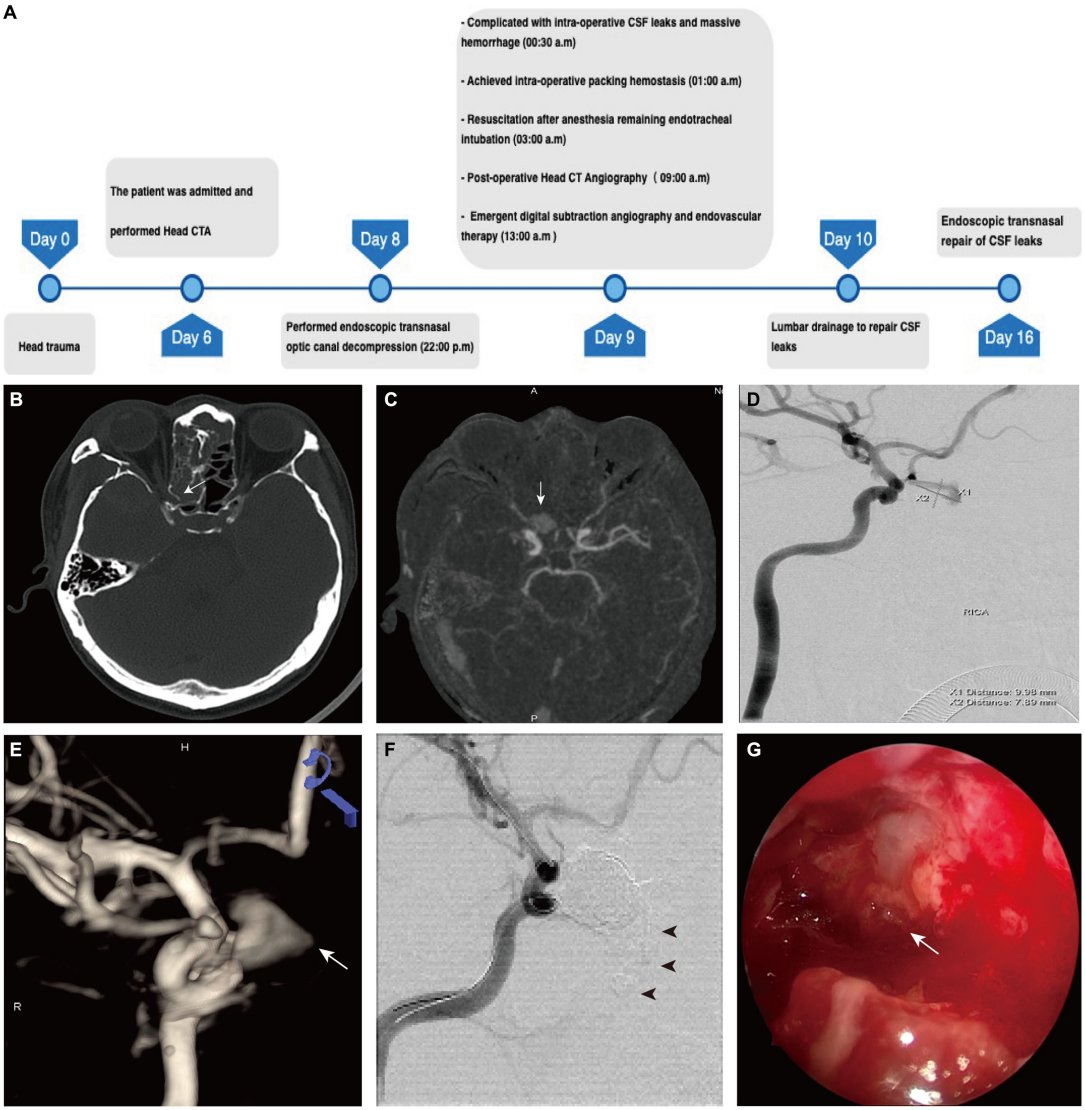
The preoperative evaluation and surgical process in Case 1. **(A)** The timeline of management in case 1. **(B)** Preoperative CT of the head demonstrates lateral wall of sphenoid sinus cavity that protrudes into the optic canal (*arrow*). **(C)** Postoperative CTA shows enhancement of the central patent portion of the pseudoaneurysm that protrudes into the sphenoid sinus cavity (*arrow*). **(D)** A pseudoaneurysm is visualized arising from the ophthalmic artery in the early arterial phase of ophthalmic artery after contrast injection. **(E)** 3D reconstruction of DSA demonstrates the irregular pseudoaneurysm with the feature of re-rupture (*arrow*). **(F)** The angiogram obtained after endovascular treatment demonstrates complete occlusion of the pseudoaneurysm. Onyx was infused into the ethmoid sinus cavity (*arrowhead*). **(G)** The surgical field of cerebrospinal fluid leak repair demonstrates the residual pulsation of embolized pseudoaneurysm (*arrow*).

A 35 year-old male patient was transferred to our hospital due to right eye visual loss after head trauma for 6 days (Figure 1A). The patient was conscious, alert, and oriented, but right direct pupillary light reflex was absent. The indirect pupillary light reflex was intact. Head computed tomography (CT) demonstrated fractures of the sphenoid bone and a slim right frontal epidural hematoma (Figure 1B). Computed tomography angiogram (CTA) ruled out contrast extravasation and pseudoaneurysm. Diagnosis of right traumatic optic neuropathy led to trans-ethmosphenoid endoscopic optic canal decompression.

During the procedure, clean cerebrospinal fluid emanated from the planum of the sphenoid bone upon removal of mucosa and bone fragments. A micro-diamond drill delicately thinned the right optic canal, and pulsatile bleeding indicated potential injury to the internal carotid artery during local drilling. Hemostasis was achieved with absorbable gelatin sponges and femoral muscle graft compression, and bilateral nasal canals were packed with gauze to secure tamponade. The patient awoke without additional neurological impair, and postoperative CTA and following cerebral angiography revealed a pseudoaneurysm arising from the right ophthalmic artery that intruding into the ethmoid sinus (Figures 1C–E).

For treatment, patient immediately underwent interventional intravascular aneurysm coiling. Using a 7 × 15 mm HyperForm balloon (eV3) in the ophthalmic segment of the internal carotid artery, a microcatheter (Echelon 10, eV3) was introduced into the aneurysm over a 0.014 microwire (Fathom14, Boston Scientific). An 18 × 50 mm Axium coil (eV3) was deployed into the aneurysm, followed by dilation of the balloon to seal the ophthalmic artery orifice. Oxyn-18 (eV3) was injected into the aneurysm under fluoroscopic guidance, with careful attention to injection rates and reflux monitoring. A small amount of glue was introduced into the ethmoid cavity during the procedure (Figure 1F). Subsequent angiography revealed no blood flow into the pseudoaneurysm, confirming patent ophthalmic artery. Gradual removal of nasal packings, monitored by angiogram, showed no contrast agent flooding the pseudoaneurysm or sinus cavity. The patient remained stable with no additional neurological symptoms.

On the second day, patient experienced cerebrospinal fluid rhinorrhea, prompting lumbar drainage at a rate of 10 mL per hour. Despite 6 days of drainage, the CSF leak persisted, leading to endoscopic repair surgery due to the sizable skull defect. The location of the CSF leak was identified as clear fluids slowly flowing from the lateral wall of the sphenoid sinus cavity. Pulsating movements beneath the skull base indicated the presence of the embolized pseudoaneurysm (Figure 1G). To minimize potential complications, the pseudoaneurysm was not fully exposed during surgery. The skull deficit was repaired using autologous fat and muscle tissue from the right thigh. Post-surgery, lumbar drainage continued for 6 days at a rate of 10 mL per hour, resulting in resolution of CSF leak upon removal of the drainage system.

At 3 month follow-up, CTA revealed no evidence of pseudoaneurysm, although the patient’s right eye remained amaurotic.

## Case 2

**Figure 2:**
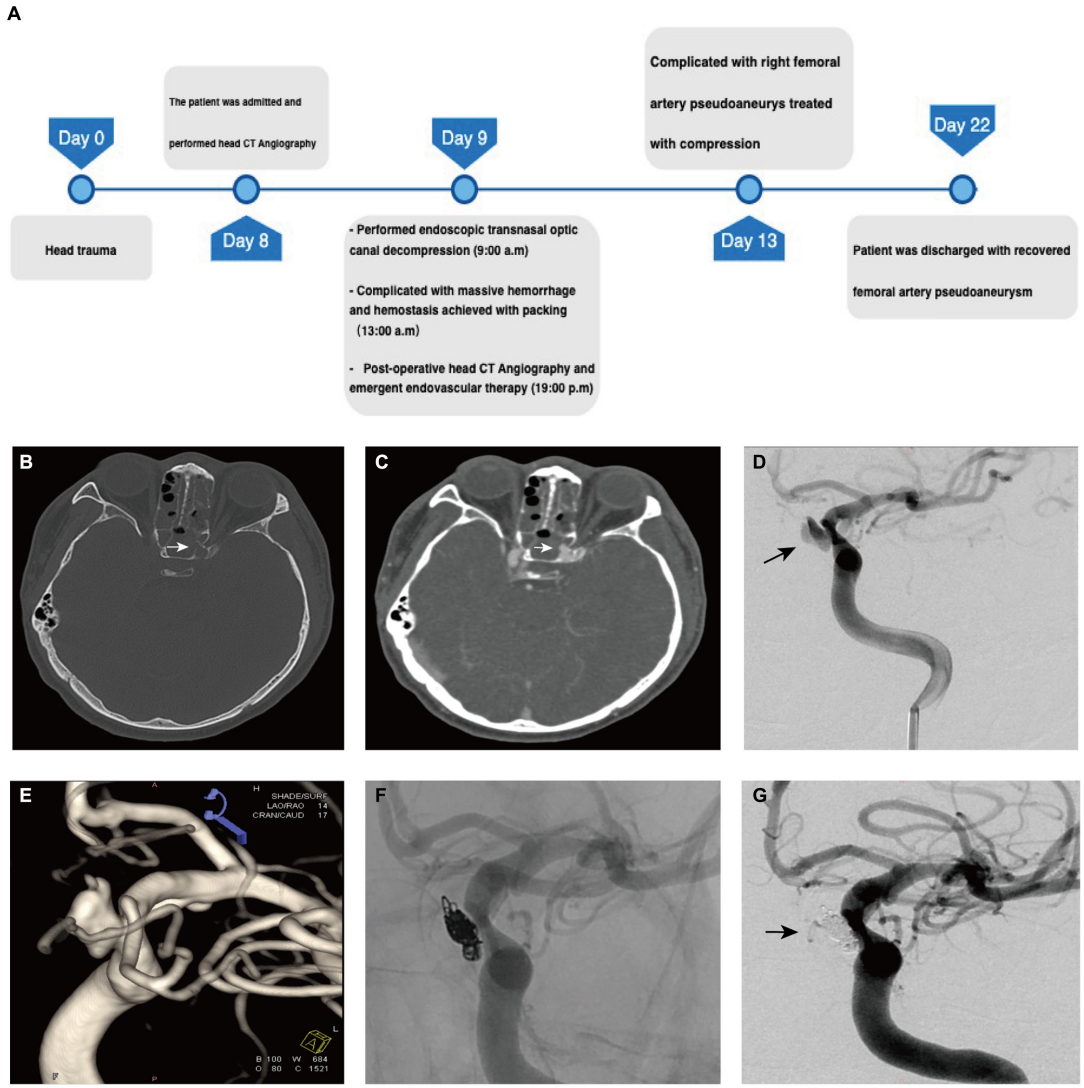
The preoperative evaluation and surgical process in Case 2. **(A)** The timeline of management in case 2. **(B)** Preoperative CT of the head demonstrates lateral wall of sphenoid sinus cavity in the left that protrudes into the optic canal (*arrow*). **(C)** Postoperative CTA shows enhancement of the central patent portion of the pseudoaneurysm that protrudes into the sphenoid sinus cavity (*arrow*). **(D)** The left internal carotid injection of the contrast demonstrates a pseudoaneurysm arising from ICA. **(E)** 3D reconstruction of DSA demonstrates the irregular pseudoaneurysm with the feature of re-rupture (*arrow*). **(F)** The angiogram obtained after endovascular treatment demonstrates complete occlusion of the pseudoaneurysm with coils. **(G)** Post-embolization angiogram demonstrates complete occlusion of the pseudoaneurysm cavity (*arrow*).

A 29 year-old male patient was transferred to our hospital following an 8 day history of visual loss after a 3 meter fall-induced head injury (Figure 2A). Upon admission, the patient was conscious, alert, and oriented, with the left eye showing no light response and loss of direct and indirect pupillary light reflexes. The vision in the right eye was measured at 0.6. Head CT revealed multiple skull base fractures, including those involving the left sphenoid, frontal, and orbit bones, as well as narrowing of the left optic nerve canal (Figure 2B). CTA showed no evidence of contrast extravasation or pseudoaneurysm. The patient underwent trans-ethmosphenoid endoscopic left optic canal decompression for left traumatic optic neuropathy.

During the procedure, significant bleeding was encountered upon removal of skull base bone fragments, suggesting potential damage to the internal carotid artery. Hemorrhage control was achieved successfully using compression with an absorbable gelatin sponge, fascia, and muscles harvested from the right thigh. Bilateral nasal canals were then packed with gauze to reinforce tamponade. The patient recovered from anesthesia without any additional neurological deficits. Emergency postoperative CT angiography revealed a dissected pseudoaneurysm originating from the ophthalmic segment of the left internal carotid artery protruding into the sphenoid sinus (Figure 2C).

Subsequently, patient underwent emergent cerebral angiography, which revealed a 5.7 × 8.8 cm ophthalmic segment aneurysm of the left internal carotid artery (Figures 2D,E). The aneurysm was initially temporally occluded using an inflated 4 × 20 mm HyperForm balloon (eV3), followed by insertion of a 5 × 20 mm coil into the aneurysm using a microcatheter (Echelon 10, eV3). Additional coils (3 × 10 mm and 1.5 × 2 mm) were deployed into the cavity before final closure of the aneurysm opening (Figure 2F). Post-procedural imaging confirmed tight coiling of the aneurysm with no detectable bloodstream flow within the sac (Figure 2G).

The patient experienced a postoperative complication in the form of a pseudoaneurysm at the puncture site of the right femoral artery, which resolved after 1 week of compression therapy. The 6 month post-operative follow-up at the local hospital revealed no recurrence of the dissected pseudoaneurysm.

## Discussion

TON presents with visual impairment following head and orbit trauma, with an incidence of 0.5–5% (3). The precise pathophysiology of TON remains elusive, though it is hypothesized that mechanical forces acting on optic nerve axons may lead to microcirculatory impairment, eventually progressing to ischemic necrosis and resultant visual loss (1, 4, 5). While consensus on TON treatment is lacking, surgical optic canal decompression is increasingly recognized as a viable approach (6). TON often coexists with craniomaxillofacial trauma (7), which can be associated with vascular complications such as traumatic carotid-cavernous fistula, traumatic carotid pseudoaneurysm, and carotid dissection (8). Although traumatic carotid injury is relatively rare (9, 10), patients with TON should undergo thorough evaluation, including computed tomographic angiography, particularly if surgical intervention is anticipated (11).

Intraoperative rupture of an internal carotid pseudoaneurysm is a critical event due to rapid high-flow hemorrhage obscuring the surgical field. Thus, meticulous preoperative preparation is essential for patient safety. In our preparation protocol, we ensured exposure and disinfection of patient’s right lateral thigh for potential repair of cerebrospinal fluid leak and compression of internal carotid injury. Gardner et al. proposed an algorithm for intraoperative management of internal carotid injury, recommending bipolar coagulation or aneurysm clip for small injuries (2–3 mm) and direct occlusion with aneurysm clip or muscle pack for large injury (>3 mm) (12). A modified protocol for iatrogenic internal carotid artery injuries in endoscopic endonasal surgery emphasizes management tailored to ICA injury category (13). However, identification of injury size and category within the confined nasal cavity amidst high-pressure hemorrhage can be challenging. Given the association of both patients with skull base fracture and the likelihood of internal carotid pseudoaneurysms, we opted for direct compression and hemostasis with autologous fascia and muscle from the lateral thigh.

Immediate angiography evaluation and treatment are imperative following intraoperative hemostasis. Flow-diverting stents offer a potential cure for internal carotid artery pseudoaneurysms by reducing blood flow into the aneurysm and providing a scaffold for vessel wall endothelialization (14, 15). Nariai et al. reported embolizing a pseudoaneurysm in the cavernous segment of the internal carotid artery following a vessel injury in transnasal endoscopic pituitary resection using Pipeline Flex (16). The safety and efficacy of Pipeline for the treatment of extracranial internal carotid artery pseudoaneurysm has been demonstrated in a multicenter retrospective study (17). However, most studies of ICA pseudoaneurysms have focused on those occurring in the cavernous segment of the ICA (16), with limited clinical research on pseudoaneurysms located in the ophthalmic artery. As the result, more research is needed on the treatment of pseudoaneurysms arising from the ophthalmic artery and the ophthalmic segment of the ICA.

It should be emphasized while covered stents provide a physical barrier isolating the pseudoaneurysm from blood flow, they may induce ischemic complications if the pseudoaneurysm arises from or is adjacent to an important branch vessel. Several cases have reported decreased blood supply to the ophthalmic artery and postoperative visual impairment following the use of Willis covered stents for treating pseudoaneurysms in the ophthalmic segment of the carotid artery (18). Consequently, covered stents may not be suitable in such cases.

Patients receiving flow-diverting stents require antiplatelet medications for at least 3 months and remain at risk of rebleeding until vessel wall reconstruction is complete. Given the possibility of re-operation to repair CSF rhinorrhea in the first case, rapid embolization of the aneurysm with coils and Onyx to prevent rebleeding becomes even more crucial. Therefore, we opted for simple embolization of the pseudoaneurysm without the application of flow diverter devices.

Notably, endovascular intervention for ophthalmic pseudoaneurysms should prioritize the protection of ophthalmic arteries to prevent retinal ischemia and associated permanent vision loss (19). Although totally occlusion of ophthalmic artery would not cause additional neurological impairment for patients already suffering from amaurosis before endovascular embolization, those with remaining eyesight still have an opportunity for partial or full recovery if right optic canal decompression is achieved. As a result, the protection of the ophthalmic artery should be a procedural focus. To mitigate the risk of Onyx reflux into patent arteries of the pseudoaneurysm, coils were placed to restrict Onyx liquid within the pseudoaneurysm sac, forming a concrete-like structure and limiting reflux into and embolization of the ophthalmic artery. Meanwhile, an inflated balloon decreased blood flow velocity and facilitated the solidification of Onyx in the pseudoaneurysm sac, preventing Onyx leakage back into patent arteries. Despite these techniques, the ophthalmic artery maintained sufficient blood flow.

Interestingly and notably, during the slow injection of Onyx into the pseudoaneurysm, it migrated into the ethmoid and sphenoid sinus cavities, indicating the vulnerability of the thin-walled pseudoaneurysm to rupture. However, the liquid properties of Onyx may mitigate wall irritation and reduce the risk of rupture during surgery. Therefore, Onyx embolization could serve as an effective method to prevent rebleeding in unstable pseudoaneurysms.

As cranial cerebrospinal fluid leakage persisted after lumbar drainage, we performed endoscopic trans-nasal surgery for repair. Observing the pulsatility of the embolized pseudoaneurysm in the medial wall of the sphenoid sinus, we opted not to extensively explore for the specific location and size of the dural defect. Instead, we utilized a complex packing comprising fat, muscle, and fascia from the left thigh to repair the defect in the lesser wing of the sphenoid bone. The advantage of fat lies in its avascular nature and low metabolic requirements, inhibiting necrosis and scar tissue formation (20). Moreover, adipose tissue releases angiogenic factors, promoting neovascularization around the skull base defect and facilitating the survival and growth of repaired tissues (21).

In the second case, the patient presented with a dissecting pseudoaneurysm in the ophthalmic segment of the internal carotid artery, which could have been better treated with a combination of coiling and flow-diverting stent placement. However, the patient opted for balloon-assisted coil embolization after through adequate communication due to personal reason. Indeed, clinical management depends on multiple factors. It is noteworthy that current reports on the use of coils and Onyx in the treatment of similar cases are concentrated in medical centers in developing countries (22). This phenomenon may be attributed to the global uneven popularity of flow diverter devices. Additionally, long-term patient follow-up should be strengthened to observe changes in dissecting aneurysms after treatment with this method and the potential recurrence of pseudoaneurysms. Unfortunately, the patient was not a local resident, and follow-up imaging data were not available.

## Conclusion

The intraoperative rupture of an ICA pseudoaneurysm represents a rare yet potentially catastrophic complication in patients with TON. We recommend preoperative angiography for all TON patients undergoing surgical optic canal decompression to identify potential vascular abnormalities. Intraoperative detection of massive bleeding should raise suspicion for ICA pseudoaneurysm rupture, necessitating immediate intervention to achieve hemostasis, which may involve muscle packing and gauze plugging of the nasal cavity. Postoperative emergency angiography and endovascular therapy are essential for evaluating and repairing cerebral vascular injuries. Additionally, endoscopic trans-nasal surgery for repairing cerebrospinal fluid leaks resistant to lumbar drainage can be both efficient and safe following pseudoaneurysm embolization. These measures collectively contribute to improved patient outcomes and reduced risks associated with this complex clinical scenario.

## Data availability statement

The original contributions presented in the study are included in the article/supplementary material, further inquiries can be directed to the corresponding author.

## Ethics statement

The studies involving humans were approved by the Ethics Committee of the Affiliated Hospital of Yunnan University. The studies were conducted in accordance with the local legislation and institutional requirements. Written informed consent for participation was not required from the participants or the participants’ legal guardians/next of kin in accordance with the national legislation and institutional requirements. Written informed consent was obtained from the individual(s) for the publication of any potentially identifiable images or data included in this article.

## Author contributions

ZY: Funding acquisition, Resources, Writing – original draft. JQ: Resources, Supervision, Writing – review & editing. LW: Methodology, Project administration, Writing – original draft. XY: Writing – review & editing. ZL: Writing – original draft. XC: Writing – review & editing. HX: Writing – review & editing. YL: Writing – review & editing. YC: Writing – review & editing. CD: Supervision, Writing – review & editing. ZG: Writing – review & editing, Conceptualization, Methodology, Supervision.
